# Roles of osteocytes in phosphate metabolism

**DOI:** 10.3389/fendo.2022.967774

**Published:** 2022-07-15

**Authors:** Toshimi Michigami

**Affiliations:** Department of Bone and Mineral Research, Research Institute, Osaka Women’s and Children’s Hospital, Osaka Prefectural Hospital Organization, Izumi, Japan

**Keywords:** phosphate, osteoblast, osteocyte, fibroblast growth factor 23, regulation

## Abstract

Osteocytes are dendritic cells in the mineralized bone matrix that descend from osteoblasts. They play critical roles in controlling bone mass through the production of sclerostin, an inhibitor of bone formation, and receptor activator of nuclear factor κ B ligand, an inducer of osteoblastic bone resorption. Osteocytes also govern phosphate homeostasis through the production of fibroblast growth factor 23 (FGF23), which lowers serum phosphate levels by increasing renal phosphate excretion and reducing the synthesis of 1,25-dihydroxyvitamin D (1,25(OH)_2_D), an active metabolite of vitamin D. The production of FGF23 in osteocytes is regulated by various local and systemic factors. *Phosphate-regulating gene homologous to endopeptidase on X chromosome* (*PHEX*), *dentin matrix protein 1* (*DMP1*), and *family with sequence similarity 20, member C* function as local negative regulators of FGF23 production in osteocytes, and their inactivation causes the overproduction of FGF23 and hypophosphatemia. Sclerostin has been suggested to regulate the production of FGF23, which may link the two functions of osteocytes, namely, the control of bone mass and regulation of phosphate homeostasis. Systemic regulators of FGF23 production include 1,25(OH)_2_D, phosphate, parathyroid hormone, insulin, iron, and inflammation. Therefore, the regulation of FGF23 in osteocytes is complex and multifactorial. Recent mouse studies have suggested that decreases in serum phosphate levels from youth to adulthood are caused by growth-related increases in FGF23 production by osteocytes, which are associated with the down-regulation of *Phex* and *Dmp1*.

## Introduction

Osteocytes, which are terminally differentiated cells of the osteoblast lineage, are dendritic cells embedded within the mineralized bone matrix (1–3). Although osteocytes are the most abundant among all cells in bone, their location and inaccessibility has delayed our understanding of their function at the molecular level. In the past few decades, mounting evidence has indicated that osteocytes play important roles in bone homeostasis. They produce sclerostin, a secreted potent suppressor of bone formation (4, 5). Furthermore, critical roles for osteocyte-derived receptor activator of nuclear factor κ B ligand (RANKL) in the control of postnatal bone resorption have been demonstrated in mouse models in which its expression was specifically deleted from osteocytes (6).

Phosphate is an essential nutrient that mediates the majority of biological processes (7). Fibroblast growth factor 23 (FGF23), which functions as a central regulator of phosphate metabolism in mammals, is mainly produced by osteocytes (8). In addition to FGF23, several other molecules responsible for phosphate homeostasis are highly expressed in osteocytes, which include *phosphate-regulating gene homologous to endopeptidase on X chromosome* (*PHEX*), *dentin matrix protein 1* (*DMP1*), and *family with sequence similarity 20, member C* (*FAM20C*), the genes responsible for hereditary hypophosphatemia (8–12). Current concepts on the molecular mechanisms by which osteocytes regulate phosphate metabolism are discussed herein.

## Osteocyte differentiation from osteoblasts

Osteocytes account for 90-95% of all bone cells in adult bone and have the longest lifespan (1–3). In the process of osteocytogenesis, a subpopulation of matrix-producing osteoblasts on the bone surface become embedded within the matrix proteins they produce and differentiate into osteocytes with a decrease in the production of the bone matrix, marked changes in morphology, and the expression of genes that constitute the signature of osteocytes (1–3). Approximately 5 to 20% of osteoblasts mature into osteocytes, while the remainder die by apoptosis or become bone lining cells (3). During the maturation of osteoblasts into osteocytes, cell morphology changes to a stellate shape with long processes. Osteocytes reside in lacunae within the mineralized bone matrix, and interconnect with each other and with osteoblasts on the bone surface by their long cytoplasmic processes running through canaliculi. As osteoblasts differentiate into osteocytes, they acquire the expression of molecules that regulate bone homeostasis and phosphate metabolism (1–3).

## Control of bone mass by osteocytes

Osteocytes embedded in the bone matrix sense mechanical signals and regulate bone formation and resorption. A previous study reported that the genetic ablation of osteocytes in mice led to osteoporotic bone loss and the suppression of mechanically-induced new bone formation (13). Sclerostin encoded by the *SOST* gene is secreted by osteocytes and suppresses bone formation (5). The inactivation and reduced expression of *SOST* in humans have been shown to be responsible for rare bone sclerosing diseases, such as sclerosteosis 1 and van Buchem diseases (14). Sclerostin binds to low-density lipoprotein receptor-related protein 5 (LRP5) and LRP6 to inhibit Wnt/ß-catenin signaling (15). Wnt/ß-catenin signaling plays a critical role in controlling bone mass by promoting the commitment of mesenchymal progenitor cells into osteoblasts as well as the proliferation and differentiation of osteoblasts. Furthermore, Wnt/ß-catenin signaling has been shown to inhibit the differentiation and activation of osteoclasts (16, 17). Mechanical signaling was found to suppress the expression of sclerostin in osteocytes, which promoted bone formation by enhancing Wnt/β-catenin signaling (4). The mechanosensor channel Piezo1 has recently been suggested to be involved in the suppression of *Sost* expression by mechanical force (18). The bone anabolic effects of parathyroid hormone (PTH) are also mediated by the down-regulation of *Sost* (19).

Osteocytes also regulate osteoclastic bone resorption by producing RANKL. Mice with the conditional deletion of RANKL from osteocytes and some mature osteoblasts exhibited markedly impaired osteoclastic bone resorption after birth, leading to the osteopetrotic phenotype (6). Therefore, osteocytes play critical roles in bone homeostasis by controlling the formation and resorption of bone in postnatal life.

## Production and effects of FGF23

As osteoblasts mature into osteocytes, they acquire the expression of various molecules involved in phosphate homeostasis, which include the genes responsible for hereditary hypophosphatemic diseases (1–3, 8). The high expression of these molecules indicates that osteocytes play essential roles in the regulation of phosphate metabolism as well as the control of bone mass.

FGF23, the key regulator in phosphate metabolism, consists of 251 amino acids including an amino-terminal signal sequence of 24 amino acids (20). It is mainly produced by osteocytes and exerts its effects on distant target organs, such as the kidneys. Its endocrine function is suggested to be conferred by its low binding affinity to heparin/heparan sulfate (21). In the kidneys, the main target for FGF23, it increases phosphate excretion by reducing the expression of type IIa and IIc sodium/phosphate (Na^+^/Pi) co-transporters (designated as NaPi-IIa and NaPi-IIc, respectively) (20). In addition, FGF23 reduces the production of 1,25-dihydroxyvitamin D [1,25(OH)_2_D], an active metabolite of vitamin D, by suppressing the expression of 25-hydroxyvitamin D 1α-hydroxylase and inducing that of 24-hydroxylase, which leads to the decreased absorption of phosphate in the intestines (20). At physiological concentrations, FGF23 requires a single-pass transmembrane protein, αKlotho as a co-receptor for its signal transduction through the FGF receptor (FGFR) (22, 23). FGF23 is inactivated by proteolytic cleavage between Arg^179^ and Ser^180^. This cleavage is prevented by the *O*-glycosylation of FGF23 at Thr^178^, a process that is mediated by UDP-*N*-acetyl-α-D-galacosamine:polypeptide *N*-acetylgalactosaminyltransferase 3 (GalNAc-T3) (24).

**Figure 1:**
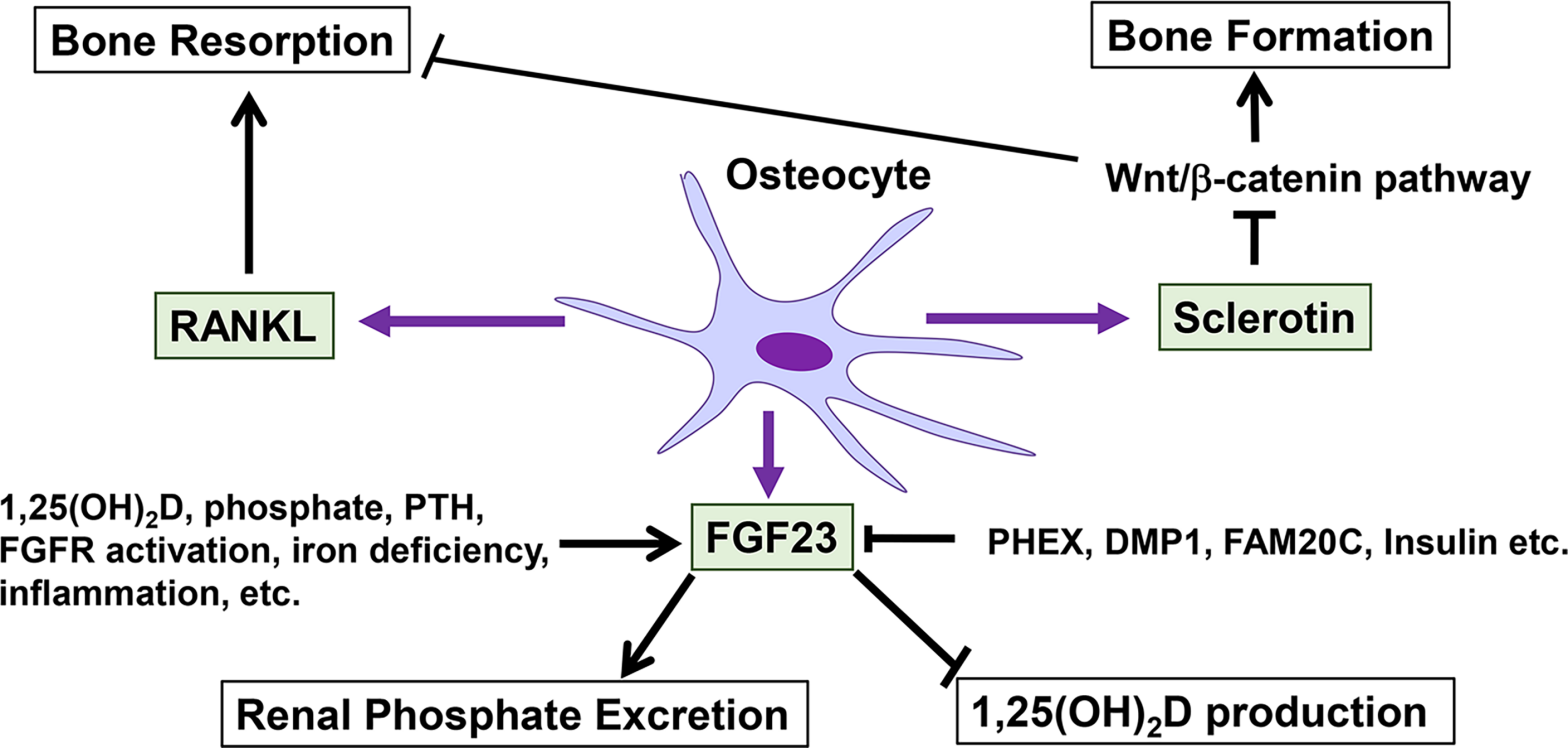
Roles of osteocytes in the control of bone mass and the regulation of phosphate metabolism. Osteocytes regulate bone formation and resorption through the production of sclerostin and RANKL. Sclerostin suppresses bone formation by inhibiting Wnt/β-catenin signaling. FGF23 produced by osteocytes plays central roles in phosphate metabolism by increasing renal phosphate excretion and decreasing the production of 1,25(OH)_2_D. The production of FGF23 in osteocytes is influenced by various positive and negative regulators.

**Figure 1** summarizes the roles of osteocytes in the control of bone mass and the regulation of phosphate metabolism.

## Local regulators of FGF23 production in osteocytes

*PHEX*, *DMP1*, and *FAM20C* are highly expressed in osteocytes (8). Since inactivating variants of these genes cause the overproduction of FGF23 in osteocytes (25), these molecules are considered to function as local negative regulators of FGF23 production in osteocytes.

The *PHEX* gene is responsible for X-linked hypophosphatemic rickets (XLH), the most common form of hereditary hypophosphatemia (9). Although the PHEX protein is suggested to function as a cell surface-bound, Zinc-dependent protease based on its structure (26), its physiological substrates remain elusive, and FGF23 does not serve as its substrate (27). *DMP1* encodes an extracellular matrix protein belonging to the small integrin-binding ligand, N-linked glycoproteins (SIBLINGs) family, and its inactivating variants cause autosomal recessive hypophosphatemic rickets type 1 (ARHR1) (10, 11).

Studies using *Phex*-deficient hypophosphatemic *Hyp* mice and *Dmp1*-null mice have suggested that enhanced FGFR signaling in osteocytes contributes to the increased production of FGF23 in XLH and ARHR1 (8, 28, 29). In *Hyp* mice, the osteocytic expression of *Fgf1*, *Fgf2*, *Fgfr1–3*, and *Egr-1*, which is a target gene of activated FGFR signaling, was found to be markedly up-regulated (8), and the osteocyte-specific deletion of *Fgfr1* partially restored the overproduction of FGF23 and attenuated hypophosphatemia and mineralization defects (29). In a culture of bone marrow stromal cells isolated from *Dmp1*-null mice, the inhibition of FGFR signaling using SU5402 prevented increases in FGF23 expression levels (28). Furthermore, mice with the transgenic overexpression of high-molecular-weight isoforms of FGF2 in osteoblast lineage cells exhibited elevated FGF23 levels and hypophosphatemic rickets (30). In humans, osteoglophonic dysplasia caused by activating variants in FGFR1 is often associated with hypophosphatemia due to elevated FGF23 levels (31). These findings support activated FGFR signaling in osteocytes increasing the production of FGF23.

The *FAM20C* gene encodes a secreted kinase that phosphorylates a broad range of substrates, including FGF23 and proteins of the SIBLINGs family, such as DMP1 (32, 33). Inactivating variants of *FAM20C* cause Raine syndrome (RNS), an autosomal recessive disease characterized by neonatal osteosclerotic bone dysplasia of an aggressive onset and poor prognosis. Patients with mild RNS may survive and manifest hypophosphatemic rickets due to elevated FGF23 levels as well as dental anomalies (12, 34). FAM20C has been shown to directly phosphorylate FGF23 on Ser^180^, which inhibits the *O*-glycosylation of FGF23 by GalNAc-T3. Therefore, the inactivation of FAM20C may increase the protein levels of intact FGF23 by reducing its cleavage (33).

Sclerostin may also function as a local regulator of FGF23. A recent study demonstrated that a treatment with an anti-sclerostin antibody reduced serum levels of intact FGF23 and increased serum phosphate levels in wild-type and *Phex*-deficient *Hyp* mice (35). While the serum intact FGF23 levels were reduced by anti-sclerostin antibody, the levels determined by C-terminal assay was found to be unchanged. Since the C-terminal FGF23 assay detects both the intact and cleaved C-terminal fragments of FGF23, the circulating C-terminal fragments of FGF23 were likely to be increased after the treatment with anti-sclerostin antibody (35). Considering a previous report demonstrating that the C-terminal fragments of FGF23 may antagonize the action of biologically active intact FGF23 (36), the elevation in serum phosphate levels following the treatment with anti-sclerostin antibody might be mediated by both the reduction in intact FGF23 levels and the increase in the C-terminal fragments. Although the regulation of FGF23 by sclerostin may be indirect and mediated by the control of bone turnover, a cell study using the osteocytic cell line IDG-SW3 suggested the direct stimulating effects of sclerostin on the synthesis of FGF23 (37). The regulation of FGF23 by sclerostin is interesting because it suggests a connection between the two important functions of osteocytes: the control of bone mass and the regulation of phosphate metabolism.

A clinical study previously demonstrated that the intravenous administration of pamidronate to patients with osteogenesis imperfecta rapidly decreased serum intact FGF23 levels (38), which suggested that bone turnover influences serum FGF23 levels. This concept was supported by a mouse study in which interleukin-1 (IL-1)-induced local bone resorption caused elevations in serum intact FGF23 levels without increasing its mRNA levels, and this elevation in FGF23 was prevented by a pre-treatment with a bisphosphonate pamidronate (39). Similarly, in nephrectomized rats with a high bone turnover renal osteodystrophy, a treatment with a bisphosphonate risedronate suppressed the elevation of serum intact FGF23 levels (40). These findings indicate that the release of FGF23 produced by osteocytes into the circulation is accelerated in association with bone resorption.

## Systemic regulators of FGF23 production in osteocytes

In addition to the local regulators described above, FGF23 production by osteocytes is influenced by various systemic factors, some of which are described herein. Among the systemic regulators of FGF23 production, 1,25(OH)_2_D appears to be a principal regulator and increases the expression of FGF23 in osteoblast lineage cells through the vitamin D receptor (VDR)-mediated transactivation of its gene (41, 42). The importance of 1,25(OH)_2_D in the regulation of FGF23 is supported by clinical observations showing that patients with vitamin D deficiency have low levels of serum intact FGF23 (43).

PTH also stimulates the production of FGF23, as suggested by the elevated serum levels of FGF23 in patients and mouse models of hyperparathyroidism (44, 45). Elevated serum levels of intact FGF23 were also reported in patients with Jansen type metaphyseal chondrodysplasia, a skeletal dysplasia caused by activating variants in PTH receptor 1 (PTH1R) (46). The importance of PTH signaling in osteocytes for the regulation of FGF23 production has been shown in mouse studies demonstrating that the constitutive activation of PTH1R in osteocytes using a *Dmp1* promoter increased the production of FGF23 (47).

Phosphate itself also regulates the production of FGF23 in osteocytes. Previous studies reported that dietary phosphate loading increased serum intact FGF23 levels in both humans and mice (48, 49). We recently showed that a 72-hour treatment of primary mouse osteocytes with high phosphate increased FGF23 production *in vitro*, and this increase occurred at the protein level rather than at the mRNA level (50). Furthermore, a treatment of osteoblast lineage cells with high phosphate up-regulated the expression of the *Galnt3* gene, which prevented the cleavage-mediated inactivation of FGF23 (51).

Recent studies demonstrated that insulin signaling suppressed the osteocytic production of FGF23 through the activation of the AKT pathway. In clinical settings, negative correlations were reported between increases in plasma insulin levels after oral glucose loading and plasma intact FGF23 levels (52). A treatment of the cultured osteoblastic cell line UMR106 with insulin and insulin-like growth factor 1 (IGF-1) also suppressed the production of FGF23 through the activation of the AKT pathway and the inhibition of forkhead box protein 1 (FOXO1) (52). We recently reported that the osteocyte-specific deletion of phosphatase and tensin homolog deleted from chromosome 10 (PTEN), the molecule that antagonizes the insulin-induced activation of AKT, resulted in a decrease in the production of FGF23 in osteocytes, a reduction in renal phosphate excretion, and the attenuation of hyperphosphatemia (53). The knockdown of PTEN expression in UMR1-6 cells decreased the expression of *Fgf23*, which was partially restored by a treatment with rapamycin, suggesting the involvement of AKT/mechanistic target of rapamycin complex 1 (mTORC1) (53). These findings suggest that the insulin- and IGF1-induced activation of AKT in osteocytes inhibit the production of FGF23 through the FOXO1 and mTORC1 pathways.

Iron deficiency increases the production of FGF23 through the hypoxia-inducible factor 1α (HIF1α)-mediated transactivation of the gene (54, 55). The activation of the HIF pathway promotes the production of the hematopoietic hormone erythropoietin, which has been shown to increase the production of FGF23 (56).

Increased serum levels of intact FGF23 may be observed in patients with inflammatory diseases (57), and various proinflammatory cytokines, such as tumor necrosis factor α, IL-1β, and IL-6, have been reported to increase the expression of *Fgf23* (58). Several mechanisms, including the activation of the HIF pathway, the involvement of NF-κB and signal transducer and activator of transcription 3, and lipocalin 2-mediated induction, have been suggested to contribute to inflammation-associated increases in the production of FGF23 (59–62).

Many other factors have also been suggested to regulate the production of FGF23, and there are several excellent review articles on this topic (57, 58). Therefore, the regulation of FGF23 is multifactorial and complex, and has not yet been elucidated in detail.

## Growth-related changes in osteocytes and alterations in phosphate metabolism

**Figure 2:**
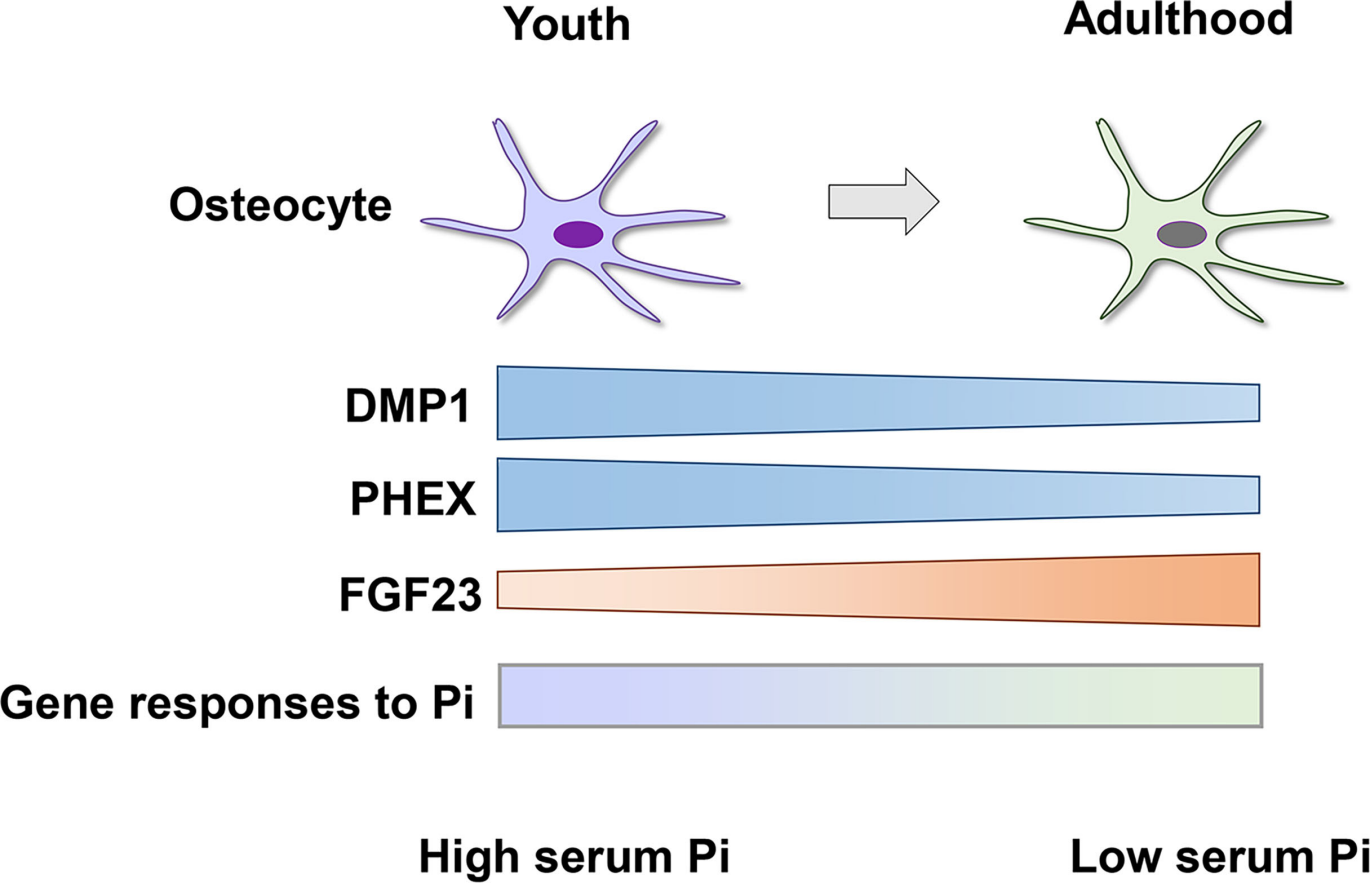
Growth-related changes in osteocytes cause a decrease in serum phosphate levels from youth to adulthood. The osteocytic expression of DMP1 and PHEX declines from youth to adulthood, which leads to an increase in the production of FGF23 and decrease in serum phosphate (Pi) levels. Gene responses of osteocytes to extracellular Pi are distinct between youth and adulthood (depicted as a shaded bar), suggesting growth-related differences in the sensitivity to the altered availability of Pi.

Serum levels of phosphate are higher in children than in adults (63), which may be due to the high need for phosphate for the growth of the skeleton and soft tissues. However, the mechanisms underlying growth-related changes in phosphate metabolism remain unclear. Since osteocytes play central roles in phosphate homeostasis, we recently investigated the relationship between growth-related skeletal changes and alterations in phosphate metabolism from youth to adulthood using young (4-week-old) and adult (12-week-old) mice (50). Although serum phosphate levels were lower in young mice, serum intact FGF23 levels and the osteocytic production of FGF23 increased from youth to adulthood and were associated with the enhancement of the FGF23-mediated-bone-kidney axis (50). An analysis of osteocytes isolated from young and adult mice revealed that the mRNA and protein levels of Dmp1 and mRNA levels of Phex declined from youth to adulthood. Since they function in the negative regulation of FGF23 production, the down-regulation of Dmp1 and Phex may be one of the mechanisms contributing to growth-related increases in the production of FGF23 and decreases in serum phosphate levels (50). In isolated osteoblasts and osteocytes, gene responses to elevated extracellular phosphate levels were also markedly altered from youth to adulthood (50). These findings provide evidence for the critical roles of osteocytes in growth-related alterations in phosphate metabolism (**Figure 2**).

## Phosphate sensing in osteocytes

To maintain phosphate homeostasis, organisms need to sense environmental and internal levels of phosphate and adapt to changes. Although the molecular mechanisms for phosphate sensing have been extensively investigated in unicellular organisms, such as bacteria and yeast, as well as in plants (64–66), the mechanisms by which mammals sense phosphate levels in individual cells or the whole body currently remain unknown. Previous studies, including ours, demonstrated that an elevation in extracellular phosphate directly exerted its effects on various cell types, including bone cells, through the activation of signaling pathways, such as the FGFR and Raf/MEK/ERK pathways (7, 67–70). The responsiveness of cells to elevated extracellular phosphate levels indicates that phosphate availability is detectable at the individual cell level. Since osteocytes play a central role in phosphate homeostasis, they may sense phosphate availability in the whole body. This concept appears to be supported by the close relationship between growth-related changes in osteocytic gene expression and their responses to phosphate and alterations in phosphate metabolism from youth to adulthood (50). A previous study reported that phosphate loading in mice up-regulated the skeletal expression of *Galnt3* by activating FGFR and increased the production of FGF23, suggesting the involvement of FGFR in phosphate sensing in mammals (51).

The parathyroid glands also respond to altered levels of extracellular phosphate. The secretion of PTH is stimulated by phosphate, and a recent study has suggested that this process is mediated by a direct action of phosphate on the calcium-sensing receptor (71). Thus, calcium-sensing receptor may function as a phosphate sensor in the parathyroids.

## Conclusion

Osteocytes embedded in the mineralized bone matrix play central roles in the regulation of phosphate metabolism as well as in the control of bone mass. They control bone formation and resorption by producing sclerostin and RANKL, respectively. FGF23 produced mainly by osteocytes functions as a key regulator of phosphate homeostasis, and it increases renal phosphate excretion and decreases the synthesis of 1,25(OH)_2_D. The production of FGF23 in osteocytes is influenced by multiple local and systemic regulators, and some of the local regulators, such as PHEX, DMP1, and FAM20C, were found to be responsible for hereditary hypophosphatemic diseases associated with the overproduction of FGF23. Serum phosphate levels are higher in children to meet the high needs for phosphate during growth. Mouse studies have suggested that decreases in serum phosphate levels from youth to adulthood are associated with growth-related increases in the production of FGF23 in osteocytes, which may be attributed to the down-regulation of PHEX and DMP1. Since osteocytes govern phosphate homeostasis, they may be responsible for sensing phosphate availability in the whole body. Although the mechanisms by which mammals sense phosphate levels remain largely unknown, FGFR appears to be involved in the process. Further clarification of the mechanisms by which osteocytes sense phosphate availability and regulate the production of FGF23 will contribute to a more detailed understanding of the pathogenesis of conditions with abnormal phosphate metabolism and the development of effective treatments.

## Author contributions

TM developed the concept and prepared the manuscript.

## Funding

Preparation of the manuscript was supported by a grant from Japan Society for the Promotion of Science (JSPS KAKENHI Grant Number 21K07835) to TM.

## Conflict of interest

The author declares that the research was conducted in the absence of any commercial or financial relationships that could be construed as a potential conflict of interest.

## Publisher’s note

All claims expressed in this article are solely those of the authors and do not necessarily represent those of their affiliated organizations, or those of the publisher, the editors and the reviewers. Any product that may be evaluated in this article, or claim that may be made by its manufacturer, is not guaranteed or endorsed by the publisher.
